# Development of a set of patient reported outcome measures for patients with benign liver tumours and cysts: patient focus groups and systematic review

**DOI:** 10.1186/s41687-022-00531-1

**Published:** 2022-12-09

**Authors:** Alicia Furumaya, Lynn E. Nooijen, Martijn P. D. Haring, Hedy A. van Oers, Marlou van Beneden, Belle V. van Rosmalen, R. Bart Takkenberg, Geert Kazemier, Marc G. Besselink, Vincent E. de Meijer, Joris I. Erdmann, the Dutch Benign Liver Tumor Group

**Affiliations:** 1 Department of Surgery, Amsterdam UMC location University of Amsterdam, Amsterdam, The Netherlands; 2 Amsterdam Gastroenterology Endocrinology Metabolism, Amsterdam, The Netherlands; 3 Department of Surgery, University Medical Center Groningen, University of Groningen, Groningen, The Netherlands; 4 Child and Adolescent Psychiatry and Psychosocial Care, Emma Children’s Hospital, Amsterdam UMC location University of Amsterdam, Amsterdam, The Netherlands; 5 Amsterdam Reproduction and Development and Amsterdam Public Health, Amsterdam, The Netherlands; 6 Department of Strategy and Policy and Care Support, Amsterdam UMC location Vrije Universiteit Amsterdam, Amsterdam, The Netherlands; 7 Department of Gastroenterology and Hepatology, Amsterdam UMC location University of Amsterdam, Amsterdam, The Netherlands; 8 Department of Surgery, Amsterdam UMC location Vrije Universiteit Amsterdam, Amsterdam, The Netherlands; 9 Cancer Center Amsterdam, Amsterdam, The Netherlands

**Keywords:** Benign liver tumours, Patient reported outcomes, Symptoms

## Abstract

**Background:**

Patient reported outcome measures (PROMs) may be useful for patients with benign liver tumours and cysts (BLTC) to evaluate the impact of treatment and/or guide shared decision making. Yet, a set of PROMs relevant to patients with BLTC is currently unavailable. In this study, we selected a PROMs set for patients with BLTC.

**Methods:**

Potentially relevant patient reported outcomes (PROs) were selected by psychologist-researchers based on keywords used or suggested by participants of two virtual focus groups meetings consisting of thirteen female BLTC patients with a median age of 50 years. Subsequently, patients were asked to report their most relevant PROs. PROMs identified by systematic literature review and computerized adaptive tests (CATs) in the Patient-Reported Outcomes Measurement Information System (PROMIS) were considered in selecting the final PROMs set to assess relevant outcomes.

**Results:**

The most important PROs were: insecurity/anxiety (11/12 patients), pain (9/12 patients), fatigue (8/12 patients), and limitations in daily life (5/12 patients). The literature review included 23 studies, which used various generic and disease-specific PROMs, often not measuring (all) relevant PROs. The final selected PROMs set included numerical rating scales for pain, two questions on overall health and quality of life and four PROMIS CATs.

**Conclusions:**

A PROMs set generically and efficiently measuring outcomes relevant for patients with BLTC was developed and may be used in future research and clinical practice.

**Supplementary Information:**

The online version contains supplementary material available at 10.1186/s41687-022-00531-1.

## Background

Benign liver tumours and cysts (BLTC) are incidentally diagnosed in approximately 8% of patients undergoing abdominal imaging for any reason [1]. Hepatocellular adenoma (HCA), focal nodular hyperplasia (FNH), hepatic haemangioma, and simple hepatic cysts are the most common BLTC [1–6]. HCA in men, HCA larger than 5 cm not regressing or even growing at follow-up, and beta-catenin mutated HCA are associated with risk of bleeding and malignant transformation warranting invasive treatment [2, 7, 8]. These specific BLTC are however rare as the incidence of HCA in general is only 3–4 per 100 000 per year [9]. All other, most frequently occurring BLTC, with reported prevalence as high as 4% (FNH), 18% (simple hepatic cysts) and 20% (hepatic haemangioma) carry a negligible risk of complications. [2, 10]

Invasive treatment should only be considered in highly selected patients [2, 10]. Minimally invasive treatment and/or experimental alternatives to (minimally invasive) surgery include transarterial (chemo-) embolization/lipidolization for HCA and hepatic haemangioma, and aspiration sclerotherapy for simple hepatic cysts [11–14]. Further investigation may be warranted to evaluate appropriate indications for treatment and the outcomes of surgery and its minimally invasive alternatives.

Patient reported outcomes (PROs) are outcomes (of treatment) related to patient’s health condition, functioning and quality of life, as directly reported by patients themselves. Patient reported outcome measures (PROMs) are the instruments or tools applied to measure PROs [15]. Selection of PROMs is based on coverage of relevant symptoms, usability and the psychometric properties of candidate PROMs [16]. The Patient-Reported Outcomes Measurement Information System (PROMIS, RRID:SCR_004718) is an initiative of the National Institute of Health (NIH) and encompasses several item banks based on item response theory. Preferably, these item banks are used as computerized adaptive tests (CATs), in which items are selected based on previously answered questions. Thus, PROMIS CATs are time-efficient and generic PROMs, *i.e.* allowing for comparison between patient groups and health populations. [17]

Aggregated PROM data can be used for research purposes, for example to assess the impact of certain treatments or the quality of health care services. In contrast, PROMs in clinical practice are assessed at the individual patient level, and may be used to screen and monitor patients, guide patient-centred consultation, and aid clinical (shared) decision making [18, 19]. It is currently unclear which PROMs are most suitable to assess outcomes in patients with BLTC both in research and clinical practice.

Therefore, this study aimed to select a PROMs set based on relevant outcomes identified from focus groups with patients, PROMs previously used in literature and available PROMIS CATs, and expert opinion.

## Methods

### Study design

A consequential study design was used in this study. First, two focus groups were undertaken to determine which PROs were most relevant to patients with BLTC. Second, PROMs identified from literature review and PROMIS CATs were assessed by healthcare professionals and the Amsterdam PROM expertise center to formulate a recommendation on a PROMs set for patients with BLTC. The study was performed according to the Declaration of Helsinki (as revised in 2013). The medical ethical committee of the Amsterdam UMC, location VUmc waived the need for ethical approval, as the study was beyond the scope of the Dutch Medical Research Involving Human Subjects Act. All patients consented for participation and publication of research results after receiving telephonic and written information on the goal, structure and processing of the focus groups.

### Identification of relevant PROs through focus groups

Adult patients diagnosed with BLTC who visited the surgery or hepatology department at the Amsterdam UMC between 2010 and 2020 were previously identified through ICD-10 code searching for a retrospective cohort study on symptoms and QoL after open/laparoscopic surgical treatment or conservative management of BLTC. Patients who had participated in this study and consented to be approached for future research were selected by random sampling and sequentially (telephonically) invited by the researchers (AF and LEN) to participate in the focus groups until there were five to eight participants in each focus group.

Two separate virtual focus group meetings were organized in June and September 2020 to identify which PROs are the most relevant to patients with BLTC. The first group included patients with HCA as some of these patients may be at risk for complications (malignant transformation and bleeding). The second group included patients with FNH, hepatic haemangioma and simple hepatic cysts, because patients with these BLTC have minimal risk of complications.

Focus groups were conducted and reported according to the Standards for Reporting Qualitative Research (SRQR) guidelines (Additional file 1: Supplementary file 1) and led by an independent and experienced focus group leader (MvB). The focus groups were commenced with an open discussion with patients about various aspects of their disease to collect different potentially relevant PROs. Then, the discussion was supplemented by questions of the focus group leader based on PROs often reported in literature, and by questions from healthcare professionals from the departments of hepatology, surgery and interventional radiology of the Amsterdam UMC and University Medical Center Groningen [11, 14]. A full overview of the focus group structure is shown in Additional file 1: Supplementary file 2.

Psychologist-researchers from the Amsterdam PROM expertise center compiled a list of potentially relevant PROs. This was performed by grouping together keywords used by participants in the focus group, while listening to the experiences of the patients. Subsequently, the compiled list of PROs was shown to patients and healthcare professionals through a web-based audience response application (Mentimeter.com, Mentimeter AB (publ), Sweden). Participants were asked to provide a top-five of PROs they would like to discuss systematically during consultation. The results of the ranking of the PROs were shown directly after all participants completed the Mentimeter. Both patients and healthcare professionals (separately) were asked to respond to the results, initiating an open discussion of the relevant PROs No new PROs emerged from this discussion.

In two focus groups, a total of thirteen female patients were included. Their median age was 50 years (IQR 38-54). Five patients with HCA, five patients with FNH, two patients with hepatic haemangioma and one patient with a simple liver cyst participated. Four patients were treated surgically. One of the HCA patients was unable to participate in the latter part of the focus group. Thus the ranking of the PROs was based on the scores of twelve patients.

### Systematic review of available PROMs

A literature review adhering to the PRISMA-guidelines for systematic reviews was performed (Additional file 1: Supplementary file 3). A literature search including MeSH and title and abstract terms related to BLTC, PROs and PROMs was performed in MEDLINE (PubMed interface), Embase (Ovid interface) and PsycInfo (Ovid interface, Additional file 1: Supplementary file 4).

Screening on title and abstract and subsequently on full text according to the in- and exclusion criteria were performed independently by two reviewers (AF and LEN). Disagreement during the process was resolved by consensus. Cohort studies and randomized clinical trials assessing PROs of patients with HCA, FNH, hepatic haemangioma or simple hepatic cysts were included. Studies including patients with polycystic liver disease were included if patients with simple hepatic cysts also formed a substantial part of the studied cohort. In case of overlapping cohorts, individual judgement was made on the study to be included based on size and detail of the reported information on PROMs. Study protocols of which results were not yet published were also included.

Case reports, case series and cohort studies including less than five patients were excluded, as were studies not primarily focusing on BLTC (e.g. evaluating all liver resections). Systematic reviews were excluded, but the reference lists of relevant reviews identified in the search were manually screened for additionally eligible articles. Of the identified studies, the following data were extracted and tabulated: author, year of publication, type of BLTC (HCA, FNH, hepatic haemangioma, simple hepatic cysts or other), number of patients included, type of treatment (surgical, interventional radiology or conservative management), and type of PROM(s) used.

### Selection of a PROMs set based on literature and expert opinion

Generic and disease-specific PROMs yielded from literature review were assessed for suitability by healthcare professionals of patients with BLTC and psychologist-researchers from the Amsterdam PROM expertise center. These were compared to CATs of PROMIS corresponding to PROs identified in the focus groups.

### Evaluation of virtual focus groups

Focus groups were conducted virtually due to restrictions caused by the COVID-19 pandemic. Prior to the pandemic, virtual focus groups were already being conducted and their potential advantages were being assessed [20]. As the ability to conduct in-person focus groups was limited, the use of virtual focus groups became more widespread and necessary. The satisfaction of the participating patients and professionals and completeness of the focus groups were assessed through a web-based questionnaire (Additional file 1: Supplementary file 5) [21]. This evaluation questionnaire addressed whether participants (patients and healthcare professionals) felt they had been able to discuss all relevant PROs. This questionnaire also included an overall satisfaction rating (1–10) and assessed whether patients and healthcare professionals would prefer a future focus group to be conducted virtually or in the hospital.

## Results

### Identification of relevant PROs through focus groups

**Fig. 1:**
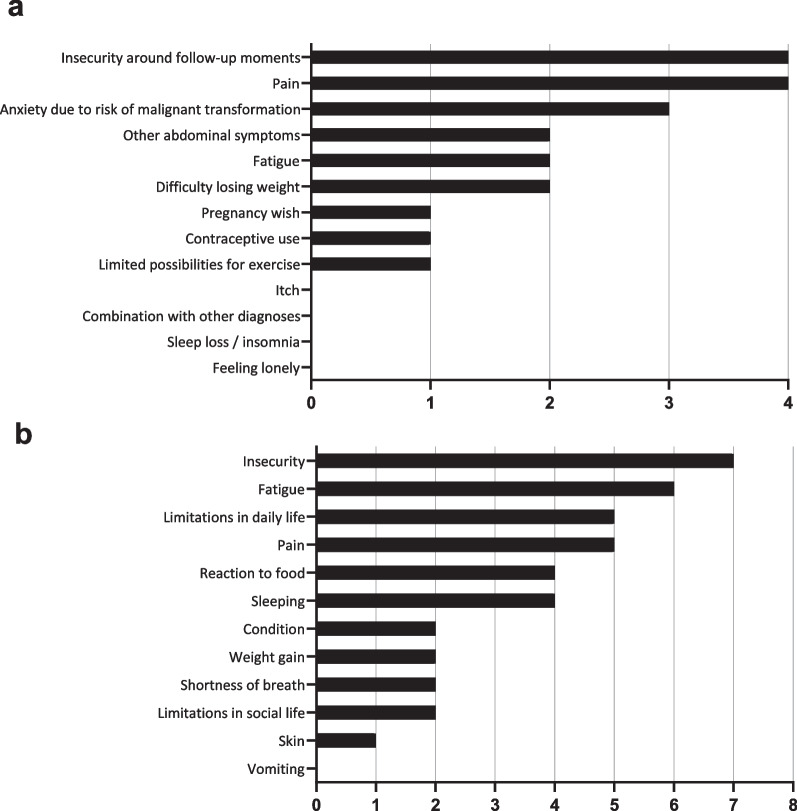
Quantitative assessment of PROs for patients with BLTC identified in the focus groups. Figures show the number of patients noting the PRO in their top-five of most important PROs. **a** In focus group 1, including patients with HCA (*n* = 4). **b** In focus group 2, including patients with FNH, hepatic hemangioma and simple hepatic cysts (*n* = 8)

A total of 13 and 12 unique PROs were identified during the two focus groups (Table 1). Top ranked PROs were insecurity/anxiety (11/12 patients), pain (9/12 patients), fatigue (8/12 patients), and limitations in daily life (5/12 patients, Fig. 1). As most PROs were independently identified as relevant in both focus groups, these were considered appropriate for all patients with BLTC.

**Table 1:** PROs identified during focus groups for patients with BLTC

PRO	Quotes
Focus group 1 (*n* = 5)	
Pain	“When the tumour was big, it did bother me, I had pain in the upper right abdomen.”
	“I had a lot of pain, but I found it difficult to determine if these symptoms could be explained by my benign liver tumour.”
	“I have stitches/pain in the right upper abdomen. Even though people say it cannot be due to my benign liver tumour, but it is the only explanation in my opinion.”
Insecurity around follow-up moments	“In the time between the check-up and getting the result, it is really on top of my mind.”
	“It’s like the sword of Damocles hanging over my head, recurring every year”
Anxiety due to risk of malignant transformation	“They told they found a liver tumour, but it was okay because it was benign. But it didn’t feel okay!”
	“Six months after my operation, I suddenly started to worry: what if it had been malignant? What if it was not fully removed, or what if it recurs?”
	Sometimes I suddenly get scared, what if it goes wrong, turns malignant and I get ill?”
Difficulty losing weight	“I was advised to lose weight, but this was very difficult because of other conditions I had.”
Fatigue	“What is still bothering me, is that I am still very tired. And there isn’t anything anybody can seem to do about it. There are still many things I want to do.”
	“There are days that I fall asleep while just sitting straight up.”
Other abdominal symptoms	“I believe I can feel the benign liver tumour every now and then, when I do specific exercises, or when doing sports or laying down in a different manner.”
Contraceptive use	“It was complicated that I could not use oral contraceptives.”
Pregnancy wish	“I know getting pregnant can cause complications. That does scare me.”
Limited possibilities for exercise	“I use to do kickboxing, but I was told to be careful.”
	“I consciously don’t play contact sports or do horse riding anymore.”
Sleep loss/insomnia	“I don’t sleep much, I get out of bed very often during the night.”
Combination with other diagnoses	“Different specialists said different things, and after a while I didn’t know whose advice to follow any more. I wish the doctors would have seen the whole me, beyond my liver (tumour).”
Pruritis	“I had an extremely itchy upper body, some evenings I needed to put myself under a cold shower.”
Feeling lonely	“I felt pretty left alone with it, it was hard to work through on a mental level”
Focus group 2 (n = 7)	
Insecurity/anxiety	“The insecurity was the major thing for me. Sometimes I feel pain in the right upper abdomen, and then I get worried the tumour might have come back after all (the operation) I went through.”
	“I still feel worried if the tumour might become malignant.”
	“I am mostly worried about the future. What if the tumour continues to grow?”
	“The main reason I feel insecure is because I don’t know which symptoms may be caused by my benign liver tumour. I have symptoms, I don’t know if they are caused by the benign liver tumour, and then I am worried that I might be exaggerating because I have these symptoms in the first place.”
Fatigue	“I am tired quite sometimes, but whether that’s related to the benign liver tumour, I don’t know. I do also have a rather busy life.”
	“I was very tired. It was a different kind of tiredness than being mentally exhausted.”
Pain	“I sometimes have a feeling of pressure when I am running, or when I sit in the car. I will just have to deal with that.”
	“I still cannot lay on the right side of my body, it feels as if something is tearing inside of me if I do.”
	“I always had stitches when exercising, but afterwards, I realized this was only on the right side of my body.”
Limitations in daily life	“I used to run twice a week, but after a while I couldn’t even walk straight because of the pain.”
Sleeping	“I don’t sleep well. This also started when I got the diagnosis.”
	“I had periods in which I couldn’t lie down, so I couldn’t sleep.”
Reaction to food	“I have very severe reactions to alcohol, I didn’t use to have that. The same goes for using paracetamol.”
	“When I have eaten anything different than usual, I feel sick and bloated.”
Limitations in social life	“I really need to divide my energy. After my operation, I couldn’t work for a whole day, or even sit up for an hour. I still have that, especially after dinner.”
Shortness of breath	“After my operation, I had persisting shortness of breath.”
Weight gain	“When I wake up, I am already tired. I used to do sports 35 h a week, but now I am tired after an hour.”
Condition	“I was very tired, I had physical difficulties doing certain types of exercise.”
Skin	“My skin is much more sensitive than it used to be.”
Vomiting	“I feel sick and vomit very easily.”

Other PROs mentioned by at least half of the patients with HCA were other abdominal symptoms (2/4) and difficulty losing weight (2/4). Other PROs mentioned by at least half of the patients with FNH, hepatic haemangioma and simple hepatic cysts were relation to food (4/8) and sleeping (4/8).

### Systematic review of available PROMs

**Fig. 2:**
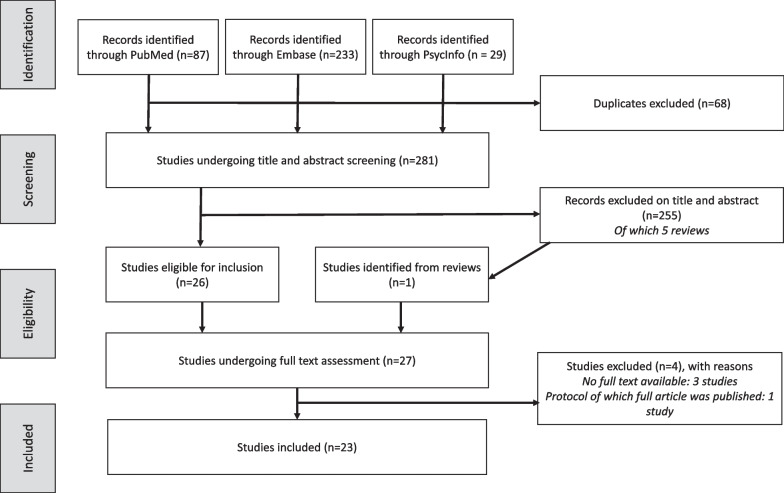
Flowchart of the study selection process for the systematic review of candidate PROMs

A total of 281 unique articles were identified in the literature review (Fig. 2). Of these, 26 original articles reported PROMs of patients with BLTC. One other study was identified by screening the references of five reviews: four were previously excluded and one was an original article combined with literature review [11, 12, 14, 22–24]. Thus, 27 original articles underwent full text assessment. One study protocol was excluded of which results were published earlier that were already included, three studies were excluded because full texts were unavailable [25–27]. Finally, 23 original studies were included [23–25, 28–46]. Two studies containing partially overlapping data were both included because one reported more PROMs, but the other included more patients [41, 42].

Of the 2952 patients included in the literature review, 1512 were diagnosed with hepatic haemangioma (51%), 629 with hepatic cysts (21%), 378 with FNH (13%), 222 with HCA (8%), and 211 with other BLTC (7%). The majority, 2220 patients (75%) underwent surgery, 675 were treated conservatively (23%) and 57 underwent an interventional radiology procedure (2%, Additional file 1: Supplementary file 6).

A total of 15 candidate PROMs were identified. Abbreviations of PROMs are explained in Table 2.

**Table 2:** Candidate PROMs for patients with BLTC identified by systematic literature review

Author (year)	Pts (*n*)	SF-36	SF-12	EQ-5D	QLQ-C30	McGill	Other	Custom
Gall (2009)	102‡	Yes§	–	–	–	–		Telephone assessment of recurrent symptoms
Loehe (2010)	99	–	–	–	–	Yes§	–	Symptom type (abdominal pain, six categories of vegetative symptoms and two categories of dyspnea) and severity (5-point scale)
Schnelldorfer (2010)	289	–	–	–	–	–	–	Subjective health status, quality of life (5-point scale), symptoms and complications
Kamphues (2011)	43	–	–	–	Yes	–		Telephone assessment of recurrent symptoms, question if patients undergo surgery again
Kneuertz (2012)	179‡	Yes§	–	–	Yes§	Yes§	QLQ-LMC21§	Subjective feelings towards surgery
Van Aalten (2012)†	50	–	Yes	Yes		–	STAI-6, IES	–
Scheuerlein (2013)	56	–	–	–	–	–		Symptoms, quality of life and medical history
Yedibela (2013)	246	–	–	Yes	–	–		–
Giuliani (2014)	75‡	Yes	–	–	–	–	–	–
Hau (2015)	100	Yes§	–	–	Yes§	Yes		–
Qiu (2015)	730	Yes	–	–	–	–		–
Klompenhouwer (2016)	48	–	Yes	–	–	–	LDSI 2.0	–
Van Rosmalen (2016)	40	–	–	–	–	Yes§	VAS pain	Change in complaints or new complaints after surgery, questions if patients would undergo surgery again
Kisiel (2017)	92	–	–	–	–	–		Telephone assessment of procedure efficacy and immediate symptom relief, clinically significant recurrent symptoms, and overall satisfaction
Perrakis (2017)	227	–	–	Yes	–	–		–
De Reuver (2018)	95	Yes	–	–	–	–	Gastrointestinal QoL index	Treatment satisfaction, persisting symptoms and additional therapy; and telephone assessment of symptoms
Janssen (2019)	88	–	–	–	Yes	–		–
Liu (2019)	205	Yes	–	–	–	–		–
Wijnands (2018) and Neijenhuis (2019)	34	Yes	–	Yes	–	–	VAS overall health, PLD-Q	Change in health on 5-point scale
Armstrong (2020)	74	–	–	–	–	–	FACT-Hep, PRISM	–
Ali Metwally (2020)	31‡	Yes	–	–	–	–	–	Body image satisfaction questionnaire
Xu (2020)	49	Yes	–	–	–	–	–	–

EQ-5D = EuroQol Five Dimensions Health Questionnaire, FACT-HEP = Functional Assessment of Cancer Therapy – Hepatobiliary, LDSI = Liver Disease Symptom Index, QLQ-LMC21 = Quality of Life questionnaire Liver Metastases Colorectal Module (by European Organisation for Research and Treatment of Cancer), McGill = McGill Pain Questionnaire, PLD-Q = polycystic liver disease questionnaire, PRISM = Patient-reported Impact of Scars Measure, pts = patients, QLQ-C30 = Core Quality of Life questionnaire (by European Organisation for Research and Treatment of Cancer), SF-12 and SF-36 = Short Form Health Survey (with 12 and 36 questions, respectively), VAS = visual analogue scale

^†^Indicates a study protocol of which the final results were not yet published

^‡^Others included were: 4 cystadeno(carcino)ma (Gall), 9 cystadenoma and 6 other not specified (Kneuertz), 48 solid and 27 cystic benign liver lesions not otherwise specified (Giuliani) and 5 cystadenoma and 3 abscesses (Ali Metwally)

^§^PROM was used in modified form

### Selection of a PROMs set based on literature and expert opinion

The candidate PROMs (generic and disease-specific) identified from literature review, and PROMIS CATs were considered for the final PROMs set. Five of the candidate PROMs were used in more than one study and all were generic. These were: SF-36 (i.e. 36-Item Short Form Survey, 10 studies), EQ-5D (4 studies), EORTC QLQ-C30 (4 studies), McGill Pain questionnaire (4 studies) and SF-12 (2 studies). Other, less frequently used generic PROMs assessed pain (VAS-score for pain), anxiety (STAI-6), and general health (VAS-score for overall health). Disease-specific PROMs were not specific to patients with BLTC, but rather specific for patients with hepatobiliary cancer (EORTC QLQ-LMC21 and FACT-HEP questionnaires) or alternate liver diseases (LDSI 2.0, Gastrointestinal QoL index and PLD-Q). Finally, PROMs were also occasionally used to assess the impact of an ultrasound (IES) or surgery (PRISM).

We evaluated which (subdomains of the) aforementioned generic PROMs identified from literature review and PROMIS CATs assessed the outcomes identified as most relevant in the focus group (i.e., insecurity/anxiety, pain, fatigue and limitation in daily life). Results are shown in Table 3. Only the SF-36 questionnaire and PROMIS CATs assessed all relevant outcomes. Due to the use of computerized adaptive testing, administering PROMIS CATs may be more relevant to patients and take up less time than the SF-36 [47, 48]. In addition, floor and ceilings effects are prevented through adding items if the coverage is not yet sufficient. [17, 49]

**Table 3:** PROMs and their (sub)domains potentially assessing relevant PROs

PROM	Items (*n*)	Relevant PROs				Other PROs
		Anxiety	Pain	Fatigue	Limitations in daily life	
SF-36	36	Mental health: 5	Bodily pain: 2	Vitality: 4	Physical functioning: 10 Role—physical: 4 Social functioning: 2 Role—emotional: 3	General health: 5 Health transition: 1
SF-12	12	Calm and peaceful: 1 Downhearted and blue: 1	–	Energy: 1	Moderate activities: 1 Climb several flights of stairs: 1 Accomplished less (physical): 1 Limited in kind of work: 1 Accomplished less (emotional): 1 Did work less careful: 1 Pain – interference: 1 Social limitations – time: 1	General health: 1
EQ-5D	5	Anxiety/depression: 1	Pain/discomfort: 1	–	Usual activities: 1	Mobility: 1 Self-care: 1
QLQ-C30	30	–	Pain: 2	Fatigue: 3	Physical functioning: 5 Role functioning:2 Emotional functioning: 4 Cognitive functioning: 2 Social functioning: 2	Global health: 2 Nausea and vomiting: 2 Dyspnea: 1 Insomnia: 1 Appetite loss: 1 Constipation: 1 Diarrhea: 1 Financial difficulties: 1
McGill	78	–	Pain: 78	–	–	–
PROMIS	NA	V1.0 Anxiety CAT	V1.1 Pain behaviour CAT V1.0 Pain intensity (NRS)	V1.0 Fatigue CAT	V1.1 Pain Interference CAT V1.2 Physical function CAT V2.0 Physical function – Mobility CAT V2.0 Ability to participate in social roles and activities CAT V2.0 Satisfaction with social roles and activities CAT	NA

If an item focused on impairment of functioning caused by anxiety, pain or fatigue, this was counted as “limitations in daily life” rather than “anxiety”, “pain” or “fatigue”

EQ-5D = EuroQol Five Dimensions Health Questionnaire, McGill = McGill Pain Questionnaire, QLQ-C30 = Core Quality of Life questionnaire (by European Organisation for Research and Treatment of Cancer), SF-12 and SF-36 = Short Form Health Survey (with 12 and 36 questions, respectively)

Therefore, to assess relevant PROs, the CATs anxiety, fatigue, pain interference and ability to participate of the Dutch-Flemish translated PROMIS® were chosen. In addition, numerical rating scales were included for current pain and worst, least and average pain over the course of a week. Two questions on overall health and general quality of life were added to assess the impact of symptoms.

### Evaluation of virtual focus groups

Eleven out of the 13 patients who participated in the focus group provided feedback, as did eight of the healthcare professionals who attended the focus group meetings and asked questions. Of the participants who provided feedback, 5/11 patients and 8/8 healthcare professionals would have preferred a virtual focus group over a focus group at the hospital if both would have been possible. Two patients preferred a focus group within the hospital, the four other patients had no preference. Patients’ median overall satisfaction score with the focus group was 8/10, and most patients reported that they had been able to tell (almost) everything (10/11 patients) they had wished to. Healthcare professionals’ median overall satisfaction score was 8.5/10, and seven out of eight healthcare professionals reported there was more than enough room for questions of healthcare professionals.

## Discussion

In the current study, we aimed to selected a set of PROMs for patients with BLTC in order to evaluate the natural course and outcomes of treatment (conservative, surgical or by interventional radiology) of patients in prospective research and to aid shared decision making. In this two-step study consisting of (1) virtual focus groups for identification of relevant PROs and (2) selection of PROMs based on literature review and expert opinion, the selected PROMs set consisted of: numerical rating scale for pain (current and most, least and average pain over a week), two questions on overall health and quality of life and PROMIS CATs for anxiety, fatigue, pain interference and ability to participate.

The coverage of relevant outcomes by both disease-specific and generic PROMs from literature and PROMIS CATs were assessed in the current study [50]. All identified disease-specific PROMs used in studies on patients with BLTC were designed for other diseases such as malignant tumours or other gastrointestinal diseases. Only the SF-36 questionnaire and PROMIS CATs had (sub)domains assessing all relevant outcomes, and both are generic. This allows for outcomes to be compared to different patient groups and the general population across different countries. [50, 51]

Both the SF-36 questionnaire and PROMIS CATs are psychometrically valid, however PROMIS CATs were selected rather than the SF-36 as PROMIS CATs carry advantages regarding their usability and psychometric properties [50]. Through the development of the PRO Rosetta Stone (PROsetta Stone®), results yielded from the PROMs set selected in the current study may still be compared to outcomes of studies using the SF-36 questionnaire. [52]

The current study has several strengths. The selection of PROMs was guided by which outcomes were most important to patients, which is essential when selecting PROMs [49]. Despite the necessity to conduct focus groups virtually, the quality of the focus group meetings was good, as measured by assessing patient satisfaction afterwards. Moreover, the quality of final selection of the PROMs set was ensured through literature review and extensive collaboration with researchers with expertise. As this was the first study exploring PROMs relevant to BLTC patients, our results could provide opportunities for future research on the PROs of patients with BLTC and the influence of treatment. Thus, selected PROMs may be readily applied in prospective studies, for example the BELIVER-study [53] (Netherlands Trial Register: NL8231), and may also provide future opportunities to identify individual patient needs and aid shared decision making [54].

The finally selected PROMs set might also have some limitations. First, the correlation between identified outcomes on which PROMs were based and the presence of BLTC is sometimes debated. We considered patients’ experiences to be decisive in the finally selected PROMs set. Nonetheless, this should be taken into consideration when applying the PROMs set. Second, translations of PROMIS CATs may not yet exist in every language and application of PROMIS CATs in more complex study designs may necessitate technological support. If CATs are not available, short forms may be used, which may be accessed through www.assessmentcenter.net/PromisForms.aspx [55]. Third, we did not include patients and experts from other countries, and only thirteen female patients participated in the focus groups. Although BLTC generally have a female preponderance, the developed set of PROMs should be validated in larger, international studies before widespread implementation [2].

## Conclusions

In conclusion, this study identified insecurity/anxiety, pain, fatigue and limitations in daily life as PROs relevant to BLTC patients through virtual focus groups. Consideration of PROMIS CATs and PROMs from literature led to a final PROMs set which is time-efficient and ensures comparability of patients with BLTC to both patients with alternate pathology as well as healthy controls. The BLTC-specific PROMs may be used for future prospective research and in clinical practice.

## Supplementary Information

**Additional file 1** Supporting information to “Development of a set of patient reported outcome measures for patients with benign liver tumours and cysts: patient focus groups and systematic review”. **Supplementary File 1:** SRQR checklist for qualitative research. **Supplementary File 2:** Focus group structure. **Supplementary file 3:** PRISMA checklist and PRISMA for Abstracts checklist for systematic reviews. **Supplementary file 4:** Search strategies. **Supplementary file 5:** Focus group evaluation questionnaires. **Supplementary file 6:** Characteristics of studies and patients identified by systematic literature review.

## Data Availability

The datasets generated or analysed during the current study are not publicly available due to privacy and ethical concerns.

## Abbreviations

BLTC: Benign liver tumours and cysts
CATs: Computerized adaptive tests
FNH: Focal nodular hyperplasia
HCA: Hepatocellular adenoma
PROMIS: Patient-Reported Outcomes Measurement Information System
PROMs: Patient reported outcome measures
PROs: Patient reported outcomes
SF-36: 36-item short form survey

## Declarations

### Disclaimers

This study was not pre-registered. The article is an original article. The article is an original work and has not been published or submitted for publication elsewhere. Preliminary findings were presented as a poster at the E-AHPBA 2021 Virtual Congress, the 14th Congress of the European-African Hepato-Pancreato-Biliary Association (E-AHPBA), 15–17 September 2021, Bilbao.
