# Supplementary material for: Development of a set of patient reported outcome measures for patients with benign liver tumours and cysts: patient focus groups and systematic review
Source: J Patient Rep Outcomes. 2022 Dec 9;6:124. doi: 10.1186/s41687-022-00531-1 (PMC9733760; doi:10.1186/s41687-022-00531-1)
Supplement: Supplementary file 1 — Additional file 1 Supporting information to “Development of a set of patient reported outcome measures for patients with benign liver tumours and cysts: patient focus groups and systematic review”. Supplementary File 1: SRQR checklist for qualitative research. Supplementary File 2: Focus group structure. Supplementary file 3: PRISMA checklist and PRISMA for Abstracts checklist for systematic reviews. Supplementary file 4: Search strategies. Supplementary file 5: Focus group evaluation questionnaires. Supplementary file 6: Characteristics of studies and patients identified by systematic literature review. [file 41687_2022_531_MOESM1_ESM.docx]

**Supporting information to “Development of a set of patient reported outcome measures for patients with benign liver tumours and cysts: patient focus groups and systematic review “**

**Table of contents**

Supplementary File 1: SRQR checklist for qualitative research 2

Supplementary File 2: Focus group structure 4

Supplementary file 3: PRISMA checklist and PRISMA for Abstracts checklist for systematic reviews 5

Supplementary file 4: Search strategies 8

Supplementary file 5: Focus group evaluation questionnaires 9

Supplementary file 6: Characteristics of studies and patients identified by systematic literature review 12

| **Section and Topic** | **Item #** | **Checklist item** | **Location where item is reported** |
| --- | --- | --- | --- |
| **TITLE AND ABSTRACT** | | |  |
| Title | 1 | Concise description of the nature and topic of the study Identifying the study as qualitative or indicating the approach (e.g., ethnography, grounded theory) or data collection methods (e.g., interview, focus group) is recommended | Title page |
| Abstract | 2 | Summary of key elements of the study using the abstract format of the intended publication; typically includes background, purpose, methods, results, and conclusions | L1-22 |
| **INTRODUCTION** | | |  |
| Problem formulation | 3 | Description and significance of the problem/phenomenon studied; review of relevant theory and empirical work; problem statement | L26-53 |
| Purpose or research question | 4 | Purpose of the study and specific objectives or questions | L53-55 |
| **METHODS** | | |  |
| Qualitative approach and research paradigm | 5 | Qualitative approach (e.g., ethnography, grounded theory, case study, phenomenology, narrative research) and guiding theory if appropriate; identifying the research paradigm (e.g., postpositivist, constructivist/ interpretivist) is also recommended; rationale** | L58-59, L67-90 |
| Researcher characteristics and reflexivity | 6 | Researchers’ characteristics that may influence the research, including personal attributes, qualifications/experience, relationship with participants, assumptions, and/or presuppositions; potential or actual interaction between researchers’ characteristics and the research questions, approach, methods, results, and/or transferability | L74-76, L78-82 |
| Context | 7 | Setting/site and salient contextual factors; rationale** | L69-85 |
| Sampling strategy | 8 | How and why research participants, documents, or events were selected; criteria for deciding when no further sampling was necessary (e.g., sampling saturation); rationale** | L67-74 |
| Ethical issues pertaining to human subjects | 9 | Documentation of approval by an appropriate ethics review board and participant consent, or explanation for lack thereof; other confidentiality and data security issues | L62-65 |
| Data collection methods | 10 | Types of data collected; details of data collection procedures including (as appropriate) start and stop dates of data collection and analysis, iterative process, triangulation of sources/methods, and modification of procedures in response to evolving study findings; rationale** | L77-85 |
| Data collection instruments and technologies | 11 | Description of instruments (e.g., interview guides, questionnaires) and devices (e.g., audio recorders) used for data collection; if/how the instrument(s) changed over the course of the study | NA |
| Units of study | 12 | Number and relevant characteristics of participants, documents, or events included in the study; level of participation (could be reported in results) | L86-90 |
| Data processing | 13 | Methods for processing data prior to and during analysis, including transcription, data entry, data management and security, verification of data integrity, data coding, and anonymization/de-identification of excerpts | L77-85 |
| Data analysis | 14 | Process by which inferences, themes, etc., were identified and developed, including the researchers involved in data analysis; usually references a specific paradigm or approach; rationale** | L77-85 |
| Techniques to enhance trustworthiness | 15 | Techniques to enhance trustworthiness and credibility of data analysis (e.g., member checking, audit trail, triangulation); rationale** | L83-85 |

# Supplementary File 1: SRQR checklist for qualitative research

| **Section and Topic** | **Item #** | **Checklist item** | **Location where item is reported** |
| --- | --- | --- | --- |
| **RESULTS** |  |  |  |
| Synthesis and interpretation | 16 | Main findings (e.g., interpretations, inferences, and themes); might include development of a theory or model, or integration with prior research or theory | L130-138 Figure 1 |
| Links to empirical data | 17 | Evidence (e.g., quotes, field notes, text excerpts, photographs) to substantiate analytic findings | Table 1 |
| **DISCUSSION** |  |  |  |
| Integration with prior work, implications, transferability, and contribution(s) to the field | 18 | Short summary of main findings; explanation of how findings and conclusions connect to, support, elaborate on, or challenge conclusions of earlier scholarship; discussion of scope of application/generalizability; identification of unique contribution(s) to scholarship in a discipline or field | L189-219 |
| Limitations | 19 | Trustworthiness and limitations of findings | L220-229 |
| **OTHER** |  |  |  |
| Conflicts of interest | 20 | Potential sources of influence or perceived influence on study conduct and conclusions; how these were managed | Title page |
| Funding | 21 | Sources of funding and other support; role of funders in data collection, interpretation, and reporting | Title page |

**The authors created the SRQR by searching the literature to identify guidelines, reporting standards, and critical appraisal criteria for qualitative research; reviewing the reference lists of retrieved sources; and contacting experts to gain feedback. The SRQR aims to improve the transparency of all aspects of qualitative research by providing clear standards for reporting qualitative research.*

***The rationale should briefly discuss the justification for choosing that theory, approach, method, or technique rather than other options available, the assumptions and limitations implicit in those choices, and how those choices influence study conclusions and transferability. As appropriate, the rationale for several items might be discussed together.*

*From:* O'Brien BC, Harris IB, Beckman TJ, Reed DA, Cook DA. Standards for reporting qualitative research: a synthesis of recommendations. Academic Medicine, Vol. 89, No. 9 / Sept 2014

DOI: 10.1097/ACM.0000000000000388

# Supplementary file 2: Focus group structure

- Introduction:
  - Aim of the focus group, i.e. selection of relevant outcomes to measure in research and clinical practice
  - Introduction of the healthcare professionals: names and department
  - Introduction of patients:
    - When were you diagnosed with a benign liver tumour or cyst?
    - How were you treated?
- Open discussion: focus group questions
  - In your experience, which factors influence(d) your quality of life?
  - Which complaints did/do you experience?
  - What are important factors to pay attention to in general?
  - Which are relevant outcomes to assess in clinical practice?
- Questions by focus group leader and healthcare professionals

| **Section and Topic** Supplementary file 3: PRISMA checklist and PRISMA for Abstracts checklist for systematic reviews | **Item #** | **Checklist item** | **Location where item is reported** |
| --- | --- | --- | --- |
| **TITLE** | | |  |
| Title | 1 | Identify the report as a systematic review. | Title page |
| **ABSTRACT** | | |  |
| Abstract | 2 | See the PRISMA 2020 for Abstracts checklist. | See below |
| **INTRODUCTION** | | |  |
| Rationale | 3 | Describe the rationale for the review in the context of existing knowledge. | L26-53 |
| Objectives | 4 | Provide an explicit statement of the objective(s) or question(s) the review addresses. | L53-55 |
| **METHODS** | | |  |
| Eligibility criteria | 5 | Specify the inclusion and exclusion criteria for the review and how studies were grouped for the syntheses. | L98-108 |
| Information sources | 6 | Specify all databases, registers, websites, organisations, reference lists and other sources searched or consulted to identify studies. Specify the date when each source was last searched or consulted. | L93-95, Suppl 3 |
| Search strategy | 7 | Present the full search strategies for all databases, registers and websites, including any filters and limits used. | Suppl 3 |
| Selection process | 8 | Specify the methods used to decide whether a study met the inclusion criteria of the review, including how many reviewers screened each record and each report retrieved, whether they worked independently, and if applicable, details of automation tools used in the process. | L96-97 |
| Data collection process | 9 | Specify the methods used to collect data from reports, including how many reviewers collected data from each report, whether they worked independently, any processes for obtaining or confirming data from study investigators, and if applicable, details of automation tools used in the process. | L108-112 |
| Data items | 10a | List and define all outcomes for which data were sought. Specify whether all results that were compatible with each outcome domain in each study were sought (e.g. for all measures, time points, analyses), and if not, the methods used to decide which results to collect. | L108-112 |
|  | 10b | List and define all other variables for which data were sought (e.g. participant and intervention characteristics, funding sources). Describe any assumptions made about any missing or unclear information. | L108-112 |
| Study risk of bias assessment | 11 | Specify the methods used to assess risk of bias in the included studies, including details of the tool(s) used, how many reviewers assessed each study and whether they worked independently, and if applicable, details of automation tools used in the process. | NA |
| Effect measures | 12 | Specify for each outcome the effect measure(s) (e.g. risk ratio, mean difference) used in the synthesis or presentation of results. | NA |
| Synthesis methods | 13a | Describe the processes used to decide which studies were eligible for each synthesis (e.g. tabulating the study intervention characteristics and comparing against the planned groups for each synthesis (item #5)). | NA |
|  | 13b | Describe any methods required to prepare the data for presentation or synthesis, such as handling of missing summary statistics, or data conversions. | NA |
|  | 13c | Describe any methods used to tabulate or visually display results of individual studies and syntheses. | L108-112 |
|  | 13d | Describe any methods used to synthesize results and provide a rationale for the choice(s). If meta-analysis was performed, describe the model(s), method(s) to identify the presence and extent of statistical heterogeneity, and software package(s) used. | NA |
|  | 13e | Describe any methods used to explore possible causes of heterogeneity among study results (e.g. subgroup analysis, meta-regression). | NA |
|  | 13f | Describe any sensitivity analyses conducted to assess robustness of the synthesized results. | NA |
| Reporting bias assessment | 14 | Describe any methods used to assess risk of bias due to missing results in a synthesis (arising from reporting biases). | NA |

| Certainty assessment | 15 | Describe any methods used to assess certainty (or confidence) in the body of evidence for an outcome. | NA |
| --- | --- | --- | --- |
| **RESULTS** | | |  |
| Study selection | 16a | Describe the results of the search and selection process, from the number of records identified in the search to the number of studies included in the review, ideally using a flow diagram. | L140-148, Fig 2 |
|  | 16b | Cite studies that might appear to meet the inclusion criteria, but which were excluded, and explain why they were excluded. | L143-146 |
| Study characteristics | 17 | Cite each included study and present its characteristics. | L146-163, Table 2 and Suppl. 5 |
| Risk of bias in studies | 18 | Present assessments of risk of bias for each included study. | NA |
| Results of individual studies | 19 | For all outcomes, present, for each study: (a) summary statistics for each group (where appropriate) and (b) an effect estimate and its precision (e.g. confidence/credible interval), ideally using structured tables or plots. | NA |
| Results of syntheses | 20a | For each synthesis, briefly summarise the characteristics and risk of bias among contributing studies. | NA |
|  | 20b | Present results of all statistical syntheses conducted. If meta-analysis was done, present for each the summary estimate and its precision (e.g. confidence/credible interval) and measures of statistical heterogeneity. If comparing groups, describe the direction of the effect. | NA |
|  | 20c | Present results of all investigations of possible causes of heterogeneity among study results. | NA |
|  | 20d | Present results of all sensitivity analyses conducted to assess the robustness of the synthesized results. | NA |
| Reporting biases | 21 | Present assessments of risk of bias due to missing results (arising from reporting biases) for each synthesis assessed. | NA |
| Certainty of evidence | 22 | Present assessments of certainty (or confidence) in the body of evidence for each outcome assessed. | NA |
| **DISCUSSION** | | |  |
| Discussion | 23a | Provide a general interpretation of the results in the context of other evidence. | NA |
|  | 23b | Discuss any limitations of the evidence included in the review. | NA |
|  | 23c | Discuss any limitations of the review processes used. | NA |
|  | 23d | Discuss implications of the results for practice, policy, and future research. | L189-235 |
| **OTHER INFORMATION** | | |  |
| Registration and protocol | 24a | Provide registration information for the review, including register name and registration number, or state that the review was not registered. | Title page |
|  | 24b | Indicate where the review protocol can be accessed, or state that a protocol was not prepared. | NA |
|  | 24c | Describe and explain any amendments to information provided at registration or in the protocol. | NA |
| Support | 25 | Describe sources of financial or non-financial support for the review, and the role of the funders or sponsors in the review. | Title page |
| Competing interests | 26 | Declare any competing interests of review authors. | Title page |
| Availability of data, code and other materials | 27 | Report which of the following are publicly available and where they can be found: template data collection forms; data extracted from included studies; data used for all analyses; analytic code; any other materials used in the review. | NA |

*From:* Page MJ, McKenzie JE, Bossuyt PM, Boutron I, Hoffmann TC, Mulrow CD, et al. The PRISMA 2020 statement: an updated guideline for reporting systematic reviews. BMJ 2021;372:n71. doi: 10.1136/bmj.n71

For more information, visit: http://www.prisma-statement.org/

| **Section and Topic** | **Item #** | **Checklist item** | **Reported (Yes/No)** |
| --- | --- | --- | --- |
| **TITLE** | | |  |
| Title | 1 | Identify the report as a systematic review. | Yes |
| **BACKGROUND** | | |  |
| Objectives | 2 | Provide an explicit statement of the main objective(s) or question(s) the review addresses. | Yes |
| **METHODS** | | |  |
| Eligibility criteria | 3 | Specify the inclusion and exclusion criteria for the review. | No |
| Information sources | 4 | Specify the information sources (e.g. databases, registers) used to identify studies and the date when each was last searched. | No |
| Risk of bias | 5 | Specify the methods used to assess risk of bias in the included studies. | NA |
| Synthesis of results | 6 | Specify the methods used to present and synthesise results. | NA |
| **RESULTS** | | |  |
| Included studies | 7 | Give the total number of included studies and participants and summarise relevant characteristics of studies. | Yes |
| Synthesis of results | 8 | Present results for main outcomes, preferably indicating the number of included studies and participants for each. If meta-analysis was done, report the summary estimate and confidence/credible interval. If comparing groups, indicate the direction of the effect (i.e. which group is favoured). | Yes/no |
| **DISCUSSION** | | |  |
| Limitations of evidence | 9 | Provide a brief summary of the limitations of the evidence included in the review (e.g. study risk of bias, inconsistency and imprecision). | No |
| Interpretation | 10 | Provide a general interpretation of the results and important implications. | Yes |
| **OTHER** | | |  |
| Funding | 11 | Specify the primary source of funding for the review. | Yes |
| Registration | 12 | Provide the register name and registration number. | NA |

*From:* Page MJ, McKenzie JE, Bossuyt PM, Boutron I, Hoffmann TC, Mulrow CD, et al. The PRISMA 2020 statement: an updated guideline for reporting systematic reviews. BMJ 2021;372:n71. doi: 10.1136/bmj.n71

For more information, visit: http://www.prisma-statement.org/

# Supplementary file 4: Search strategies

**Table S1. Search in MEDLINE (PubMed interface):** executed on 28-04-2021

| **#** | **Search terms** | **Results** |
| --- | --- | --- |
| 1 | ("Liver"[Mesh] OR hepatic[tiab] OR liver[tiab]) AND ("Cysts"[Mesh] OR “cyst”[tiab] OR “cysts”[tiab]) NOT polycystic[ti] | 11878 |
| 2 | "adenoma, liver cell"[MeSH Terms] OR “liver cell adenoma”[tiab] OR “liver cell adenomas”[tiab] OR hepatocellular adenoma*[tiab] OR “liver adenoma”[tiab] OR “liver adenomas”[tiab] OR “hepatic adenoma”[tiab] OR “hepatic adenomas”[tiab] | 2945 |
| 3 | "focal nodular hyperplasia"[MeSH Terms] OR focal nodular hyperplasia*[tiab] | 2455 |
| 4 | focal lesion*[tiab] AND benign*[tiab] AND liver[tiab] | 163 |
| 5 | ("hemangioma"[MeSH Terms] OR hemangioma*[tiab] OR haemangioma*[tiab]) AND ("Liver Neoplasms "[Mesh] OR liver[tiab] OR hepatic[tiab]) | 6144 |
| 6 | “Quality of life”[MeSH Terms] OR “Patient Reported Outcome Measures”[MeSH Terms] OR HRQOL[tiab] OR QOL[tiab] OR “quality of life”[tiab] OR “life quality”[tiab] OR “patient reported outcome”[tiab] OR “patient reported outcomes”[tiab] OR “patient reported outcome measure”[tiab] OR “patient reported outcome measures” | 370806 |
| 7 | #1 OR #2 OR #3 OR #4 OR #5 | 21352 |
| 8 | #6 AND #7 | 87 |

**Table S2. Search in Embase Classic + Embase (Ovid interface):** executed on 28-04-2021

| **#** | **Search terms** | **Results** |
| --- | --- | --- |
| 1 | liver cyst/ or ((hepatic or liver) adj3 cyst*).ti,ab,kw. NOT polycystic.ti. | 12355 |
| 2 | liver adenoma/ or (liver cell adenoma* or hepatocellular adenoma* or liver adenoma* or hepatic adenoma*).ti,ab,kw. | 5252 |
| 3 | nodular hyperplasia/ or focal nodular hyperplasia*.ti,ab,kw. | 5338 |
| 4 | (exp haemangioma/ or (haemangioma* or haemangioma*).ti,ab,kw.) and (exp liver tumour/ or liver.ti,ab,kw. or hepatic.ti,ab,kw.) | 7947 |
| 5 | (lesion* and benign* and liver).ti,ab,kw. | 6400 |
| 6 | patient-reported outcome/ or quality of life/ or (HRQOL or QOL or quality of life or life quality or patient reported outcome* or patient reported outcome measure*).ti,ab,kw. | 676715 |
| 7 | 1 or 2 or 3 or 4 or 5 | 31597 |
| 8 | 6 and 7 | 233 |

**Table S3. Search in PsycInfo (Ovid interface):** executed on 28-04-2021

| **#** | **Search terms** | **Results** |
| --- | --- | --- |
| 1 | ((hepatic or liver) adj3 cyst*).mp. NOT polycystic.ti. | 21 |
| 2 | (liver cell adenoma* or hepatocellular adenoma* or liver adenoma* or hepatic adenoma*).mp. | 2 |
| 3 | focal nodular hyperplasia*.mp. | 2 |
| 4 | (haemangioma* or haemangioma*).mp. and (liver/ or liver.mp. or hepatic.mp.) | 1 |
| 5 | (lesion* and benign* and liver).mp. or (Benign Neoplasms/ and (liver/ or liver.mp. or hepatic.mp)) | 4 |
| 6 | 1 or 2 or 3 or 4 or 5 | 29 |

# Supplementary file 5: Focus group evaluation questionnaires

**Questionnaire for patients**

*Part 1 Technical aspects*

1. I could hear the other participants well
   - Strongly disagree
   - Disagree
   - Neutral
   - Agree
   - Strongly agree
2. I could see the other participants well
   - Strongly disagree
   - Disagree
   - Neutral
   - Agree
   - Strongly agree
3. Did you experience any technical issues before or during the virtual focus group?
   - Yes
   - No

If so, which technical issues?

1. Did you find it easy to submit your top-five outcomes?
   - No, very difficult
   - No, difficult
   - Neutral
   - Yes, easy
   - Yes, very easy

*Part 2 Focus group quality*

1. I felt that I have discussed all outcomes and issues that are important to me
   - Strongly disagree
   - Disagree
   - Neutral
   - Agree
   - Strongly agree

Comments (not required):

1. The interaction with the other patients was pleasant
   - No, very unpleasant
   - No, unpleasant
   - Neutral
   - Yes, pleasant
   - Yes, very pleasant

Comments (not required):

1. The interaction with the participating healthcare professionals was pleasant
   - No, very unpleasant
   - No, unpleasant
   - Neutral
   - Yes, pleasant
   - Yes, very pleasant

Comments (not required):

1. Due to the COVID-19 pandemic, we organized the focus group sessions virtually. Before the pandemic, these sessions were generally organized at the hospital. If I would ever participate in another focus group, I would prefer
   - A virtual focus group session (the current focus group)
   - Neutral
   - A focus group session in the hospital
2. Please rate your overall satisfaction with the focus group (0-10 scale rating, 0 – not satisfied at all – up to 10 – very satisfied)

**Questionnaire for healthcare professionals**

*Part 1 Technical aspects*

1. I could hear the other participants well
   - Strongly disagree
   - Disagree
   - Neutral
   - Agree
   - Strongly agree
2. I could see the other participants well
   - Strongly disagree
   - Disagree
   - Neutral
   - Agree
   - Strongly agree

*Part 2 Focus group quality*

1. I had the impression that the participating patients were able to discuss all outcomes and issues that are important to them

- Strongly disagree
- Disagree
- Neutral
- Agree
- Strongly agree

Comments (not required):

1. I had the impression that the interaction with the healthcare professionals was pleasant for the participating patients
   - No, very unpleasant
   - No, unpleasant
   - Neutral
   - Yes, pleasant
   - Yes, very pleasant

Comments (not required):

1. I was able to discuss all outcomes and issues that I feel are important
   - Strongly disagree
   - Disagree
   - Neutral
   - Agree
   - Strongly agree

Comments (not required):

1. I felt the focus groups were sufficiently thorough, so when organizing or participating in future focus groups, I would be confident in these being conducted virtually
   - Strongly disagree
   - Disagree
   - Neutral
   - Agree
   - Strongly agree
2. If I would participate in another focus group, I would prefer
   - A virtual focus group session (the current focus group)
   - Neutral
   - A focus group session in the hospital

Please rate your overall satisfaction with the focus group (0-10 scale rating, 0 – not satisfied at all – up to 10 – very satisfied)

# Supplementary file 6: Characteristics of studies and patients identified by systematic literature review

| **Author (year)** | **Total pts (*n*)** | **BLTC (*n*)** | | | | | **Treatment (*n*)** | | **Pts with PROMs available (*n*)** |
| --- | --- | --- | --- | --- | --- | --- | --- | --- | --- |
|  |  | Cyst | Hem | HCA | FNH | PCLD | Yes§ | No |  |
| Gall (2009) | 102‡ | 67 | - | - | - | 31 | 102 | - | 64 |
| Loehe (2010) | 99 | 77 | - | - | - | 22 | 99 | - | 65 |
| Schnelldorfer (2010) | 289 | - | 289 | - | - | - | 56 | 233 | 289 |
| Kamphues (2011) | 43 | 36 | - | - | - | 7 | 43 | - | 31 |
| Kneuertz (2012) | 179‡ | 64 | 35 | 30 | 35 | - | 179 | - | 179 |
| Van Aalten (2012)† | 50 | **-** | **-** | 50 | - | - | - | 50 | 50 |
| Scheuerlein (2013) | 56 | 52 | - | - | - | 4 | 56 | - | 56 |
| Yedibela (2013) | 246 | - | 246 | - | - | - | 103 | 143 | 246 |
| Giuliani (2014) | 75‡ | - | - | - | - | - | 75 | - | 66 |
| Hau (2015) | 100 | - | - | - | 100 | - | 100 | - | 57 |
| Qiu (2015) | 730 | - | 730 | - | - | - | 730 | - | 697 |
| Klompenhouwer (2016) | 48 | - | - | 48 | - | - | - | 48 | 48 |
| Van Rosmalen (2016) | 40 | 4 | 4 | 20 | 12 | - | 40 | - | 40 |
| Kisiel (2017) | 92 | 92 | - | - | - | - | 92 | - | 48 |
| Perrakis (2017) | 227 | - | - | - | 227 | - | 93 | 134 | 189 |
| De Reuver (2018) | 95 | 86 | - | - | - | 9 | 46 | 49 | 55 |
| Janssen (2019) | 88 | 75 | - | - | - | 13 | 88 | - | 88 |
| Liu (2019) | 205 | - | 205 | - | - | - | 205 | - | 205 |
| Wijnands (2018) and Neijenhuis (2019) | 34 | 11 | - | - | - | 23 | 34 | - | 34 |
| Armstrong (2020) | 74 | - | - | 74 | - | - | 56 | 18 | 74 |
| Metwally (2020) | 31‡ | 16 | 3 | - | 4 | - | 31 | - | 31 |
| Xu (2020) | 49 | 49 | - | - | - | - | 49 | - | 49 |
| Total | 2952 | 629 | 1512 | 222 | 378 | 109 | 2277 | 675 | 2661 |
| Abbreviations: BLTC = benign liver tumours and cysts, pts = patients, Cyst = simple hepatic cyst, FNH = focal nodular hyperplasia, HCA = hepatocellular adenoma, Hem = hepatic haemangioma, PCLD = polycystic liver disease  † Indicates a study protocol of which the final results were not yet published  ‡ Others included were: 4 cystadeno(carcino)ma (Gall), 9 cystadenoma and 6 other not specified (Kneuertz), 48 solid and 27 cystic benign liver lesions not otherwise specified (Giuliani) and 5 cystadenoma and 3 abscesses (Metwally)  § All patients underwent surgery, expect 34 patients in the studies by Wijnands and Neijenhuis *et al.* and 23 patients in the study by Armstrong *et al.*, who underwent interventional radiology procedures | | | | | | | | | |
